# Seroprevalence and altitude-dependent patterns of *Toxoplasma gondii* infection in livestock from northern Armenia

**DOI:** 10.1371/journal.pone.0347101

**Published:** 2026-04-16

**Authors:** Ruzanna Gevorgyan, Sargis A. Aghayan, Oleg Shcherbakov, Ahmad Daryani, Tigran Abgaryan, Hasmik Gevorgyan

**Affiliations:** 1 Laboratory of Molecular Parasitology, Scientific Center of Zoology and Hydroecology of NAS RA, Yerevan, Armenia; 2 Research Institute of Biology, Yerevan State University, Yerevan, Armenia; 3 Toxoplasmosis Research Center, Communicable Diseases Institute, Mazandaran University of Medical Sciences, Sari, Iran; Independent Researcher, UNITED KINGDOM OF GREAT BRITAIN AND NORTHERN IRELAND

## Abstract

Toxoplasmosis, caused by the intracellular protozoan parasite *Toxoplasma gondii*, is a zoonotic disease affecting almost all warm-blooded vertebrates. Despite its importance to both humans and animals, research on toxoplasmosis in Armenia has been limited, with a notable 35-year gap in studies focused on host animals. This study aimed to conduct a survey of *T. gondii* seroprevalence among livestock in Armenia. A total of 1996 serum samples of sheep, pigs, and cattle were collected from the northern provinces (Shirak, Lori, Tavush) of Armenia and screened by the ELISA test for the presence of total anti-*Toxoplasma* IgG and IgM antibodies. In the present study, the overall seroprevalence of T. gondii in livestock from northern Armenia was 15.5% (308/1,982; 95% CI: 14.0–17.2). The overall prevalence of infection among sheep was 22.5% (196/870; 95% CI: 19.8–25.5), pigs 28.6% (4/14; 95% CI: 8.4–58.1), and cattle 10.1% (112/1,112; 95% CI: 8.4–12.0). After excluding pigs due to insufficient sample size, a Pearson’s chi-square test demonstrated a strong and statistically significant association between host species and *T. gondii* infection status (χ² = 58.31, df = 1, p < 0.001). To investigate environmental determinants of infection, species-specific logistic regression models were fitted using temperature and elevation as continuous predictors. In cattle, none of the environmental variables showed statistically significant associations with seropositivity, and model fit did not improve over the null model. In contrast, sheep displayed a strong and non-linear relationship between seropositivity and elevation. The likelihood of infection decreased from low to mid altitudes and rose slightly at the highest elevations. Temperature showed no detectable effect in either species. These findings reveal clear species-specific differences in environmental sensitivity and highlight elevation as a key ecological factor shaping *T. gondii* exposure risks in sheep.

## Introduction

Toxoplasmosis, caused by the intracellular protozoan parasite *Toxoplasma gondii*, is a widespread disease affecting almost all warm-blooded animals, with cats as the definitive hosts responsible for shedding environmentally resistant oocysts. These oocysts persist under favorable conditions and contribute to transmission [1]. The WHO classifies *T. gondii* as a major foodborne pathogen, causing severe health issues, especially in immunocompromised individuals and fetuses [2,3]. In the U.S., *T. gondii* accounts for about 24% of foodborne illness deaths [4]. It also impacts livestock, causing reproductive losses and serving as a reservoir for human infection via undercooked meat [5]. Toxoplasmosis is a significant cause of abortion in sheep and goats, leading to economic losses [6].

Epidemiological investigations in livestock are therefore essential for understanding how environmental, husbandry, and biological factors interact to shape infection patterns at the population level. Such studies provide baseline data for assessing food-safety risks, identifying high-risk production systems, and guiding targeted interventions to reduce transmission. Recent reviews emphasize that small ruminants consistently show higher seroprevalence than other food-producing species and play a disproportionate role in meat-borne human infection [7]. Epidemiological mapping of infection in different agro-ecological zones also helps clarify how altitude, climate, grazing systems, and cat density contribute to exposure, as demonstrated in various regional studies of sheep and goats [8]. Beyond animal health, such surveillance has become a public-health priority: EFSA has highlighted foodborne biological hazards, including protozoan parasites such as *T. gondii*, within its emerging-risk activities, underscoring the need for improved field data from livestock production systems to support risk-based food-safety frameworks [9].

Accurate diagnosis of *T. gondii* infection in livestock relies on a range of serological and molecular tools, each differing in sensitivity, specificity, and suitability for field epidemiology. Molecular assays such as PCR can detect active infection and parasite DNA in tissues or blood, but their sensitivity in chronically infected livestock is often limited due to low and intermittent parasitemia, making them less practical for large-scale surveillance [8,10]. By contrast, serological assays remain the cornerstone of epidemiological studies because antibodies persist for long periods following exposure, providing a reliable indicator of past infection at the population level [11,12]. Among available serological tests, ELISA is widely regarded as one of the most efficient methods for screening livestock due to its high throughput, reproducibility, and suitability for processing large sample numbers with relatively low cost and labour requirements [7,13]. ELISA platforms also demonstrate strong concordance with reference assays such as the modified agglutination test (MAT) or indirect fluorescence antibody test (IFAT), especially in sheep and goats, where serology is considered the preferred diagnostic approach for herd-level investigations [14,15].

Research on *T. gondii* in Armenia is limited but spans decades, revealing varied seroprevalence across species and regions. Initial studies in the 1960s reported low seropositivity, with humans at 3.43% and animals ranging from 0.7% to 7.7% [16–19]. Later studies in the 1970s showed higher rates, with regional differences: forest-steppe zones had the highest prevalence (up to 10%) compared to lowlands [20]. By the 1980s, seroprevalence rose significantly, particularly among cattle (32–42%), pigs (5–12%), and sheep (29%) [21,22]. Recent research (2021–2022) in Tavush Province recorded the highest seroprevalence in sheep (39%), followed by pigs (28.6%) and cattle (8.9%) [23]. These findings highlight increasing prevalence over time and geographic variation. Additionally, it was shown that the prevalence of *T. gondii* in wild birds (Passerines) captured in the south (Meghri region) of Armenia was 12% [24]. A 2018 study found *T. gondii* DNA in 10.9% of small mammals (rodents and shrews) from six different localities in Armenia [25]. The summarized results of studies on *Toxoplasma gondii* from 1961 to 2023 in Armenia are presented in the article by Daryani et al., 2025 [26].

In epidemiological studies, serological methods are favored for diagnosing *T. gondii* infection in animals because other techniques are not practical for analyzing large sample sizes [1,27].

The objective of our study was to assess the current prevalence of *T. gondii* among the main livestock species that are used for human consumption in Armenia and can have epidemiological and veterinary importance; to identify the environmental factors influencing its distribution, and to compare current seroprevalence estimates with data collected 35 years ago in order to evaluate temporal changes and address the existing gap in long-term epidemiological information for this region.

## Materials and methods

### Study site and sampling

**Fig 1:**
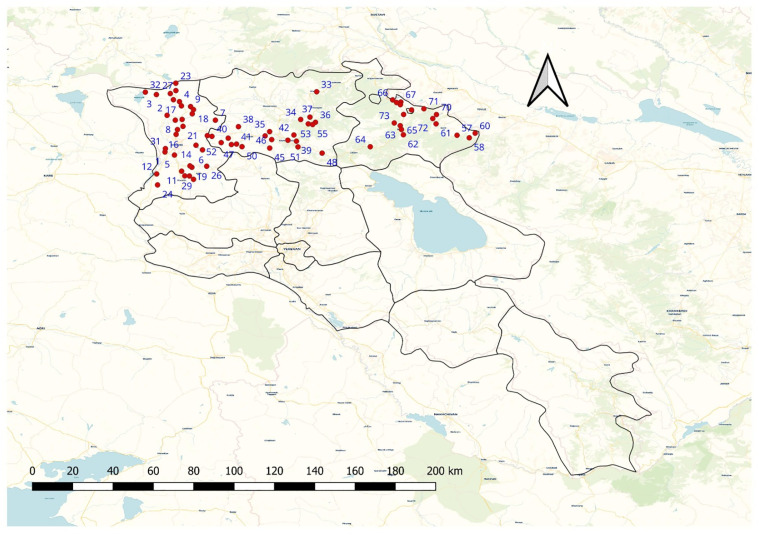
Geographical distribution of sampling sites.1-32 Shirak province: 1-Akhurik, 2-Amasia, 3-Ardenis, 4-Ashotsk, 5-Azatan, 6-Basen, 7-Dzorashen, 8-Gogahovit, 9-Hartashen, 10-Haykasar, 11-Hayrenyats, 12-Isahakyan, 13-Karmravan, 14-Karnut, 15-Keti, 16-Mayisyan, 17-Mets Sepasar, 18-Musayelyan, 19-Nor Kyank, 20-Pemzashen, 21-Pokrashen, 22-Salut, 23-Saragyugh, 24-Sarakap, 25-Sizavet, 26-Spandaryan, 27-Tavshut, 28-Torosgyugh, 29-Tufashen, 30-Vardakar, 31-Voskehask, 32-Zorakert; 33-55 Lori province: 33-Alaverdi, 34-Antaramut, 35-Arjut, 36-Debed, 37-Dzoragyugh, 38-Gogaran, 39-Gugark, 40-Hartagyugh, 41-Jrashen, 42-Karaberd, 43-Katnajur, 44-Khnkoyan, 45-Lernajur, 46-Lernapat, 47-Lernavan, 48-Margahovit, 49-Nor Khachakap, 50-Saramej, 51-Shahumyan, 52-Tsakhkaber, 53-Vahagni, 54-Vanadzor, 55-Yeghegnut; 56-73 Tavush province: 56-Aknaghbyur, 57-Artsvaberd, 58-Aygedzor, 59-Azatamut, 60-Chinari, 61-Chinchin, 62-Gandzakar, 63-Getahovit, 64-Haghartsin, 65-Ijevan, 66-Kirants, 67-Sarigyugh, 68-Sevkar, 69-Tsaghkavan, 70-Varagavan, 71-Vazashen, 72-Verin Tsaghkavan, 73-Yenokavan. The map was created using QGIS 3.40.1-Bratislava. Administrative boundary layers were obtained from Natural Earth (https://www.naturalearthdata.com/). The base map uses OpenStreetMap (OSM) data rendered with the CARTO Voyager basemap. OSM data are © OpenStreetMap contributors and are available under the Open Database License (ODbL). The CARTO Voyager basemap is provided under the Creative Commons Attribution 3.0 license.

Between April and December 2021–2023, a total of 73 localities across the three northern provinces (Shirak, Lori, Tavush) of Armenia were included in the survey. Sampling points were distributed as follows: 32 in Shirak, 18 in Tavush, and 23 in Lori province (Fig 1).

In two of the mentioned provinces, the number of sampling sites is higher due to the higher level of development of animal husbandry in these provinces. In total, serum samples from 1112 cattle, 870 sheep and 14 pigs were tested. The number of sampled animals per locality varied, but the complete locality-level distribution of sample sizes for each species is provided in S1 Table. In each province, visits were made to regional centers of the Food Safety Inspection Body of the Republic of Armenia (FSIB). The blood samples were obtained from the regional branches of the Food Safety Inspection Body of the Republic of Armenia, the Ministry of Economy, according to a collaboration request N2459-124 by the Scientific Center of Zoology and Hydroecology, NAS RA. The staff of the research team did not participate directly in the blood collection from animals. No anesthesia, euthanasia, or any kind of animal sacrifice was part of the study.

Data concerning age, gender, and breed were not available from FSIB.

The northern provinces make up about one-third of Armenia’s territory. They are located at an altitude of 380–4,090 m above sea level and border Turkey to the west, Georgia to the north, and Azerbaijan to the east. The average annual temperature ranges from −13 to −14°C in winter and +20 to +26°C in summer, and the average annual precipitation is 450–900 mm (500–1200 mm according to some sources). The Lori and Tavush provinces are considered the most humid in Armenia [28,29].

For each locality, blood samples were collected from animals from farms and households. Blood samples were collected from the jugular vein of livestock animals by trained personnel using sterile needles and plain vacutainer tubes. Blood samples were subjected to routine screening procedures at FSIB laboratories prior to being provided for the purposes of the present study. These samples were left in the laboratory for 30–60 min at room temperature for clotting. After complete clot formation, blood samples were centrifuged at 1,500–2,000 × g for 10–15 minutes at room temperature. The obtained serum was carefully collected using a sterile pipette and transferred into sterile Eppendorf tubes, which were maintained at 4°C in insulated containers with ice packs and transferred to our laboratory. Upon arrival at the laboratory, samples were either processed immediately or stored at −18°C until further analysis. A total of 1,996 serum samples were collected.

### Serological testing

The research was carried out using enzyme-linked immunosorbent assay (ELISA). Measurement of the level of total anti-*Toxoplasma* antibodies IgG and IgM in the samples was carried out using ELISA kits (ELISA kit “Toxo-antibodies-IЕA-Zoo-BEST” catalog number: V1710, Russia) according to the manufacturer’s protocol. This solid-phase ELISA detects total antibodies against *T. gondii* using a *Toxoplasma* antigen conjugated with Horseradish Peroxidase (HRP) and TMB (tetramethylbenzidine) as a substrate. The kit does not differentiate between IgG and IgM isotypes.

The samples were loaded into a 96-well plate. In any two wells (for example A1, B1), a negative control was added, and in one well (for example C1), a positive control was added, both provided by the manufacturer and were used to validate each test. 93 samples were tested simultaneously. The results were expressed as optical density (OD), absorbance was read at 450 nm with an ELISA Plate reader (BIOBASE ELISA Microplate Reader BK-EL10A). Results were interpreted based on cut-off values provided by the manufacturer. The results of the studies were taken into account only when the optical density value in the wells containing the negative control (K^-^) was not higher than 0.2 and the positive control (K^+^) was not lower than 0.5.

Critical optical density was calculated using the following formula:


$${\text{OD}}_{\text{crit}.}={\text{OD}}_{{\text{averageK}}^{-}}+\text{0}.\text{2}$$

The analysis result was considered positive if ОD_Sample_ ≥ ОD_Crit._
The analysis result was considered negative if ОD_Sample_ < ОD_Crit._
OD_Sample_ – optical density of the sample under study.

### Data analysis

We obtained climate and environmental data (temperature, rainfall (mm), rainfall intensity (mm/day), and relative humidity) from NASA Prediction Of Worldwide Energy Resources (POWER) | Data Access Viewer (DAV) v2.3.6 (https://power.larc.nasa.gov/data-access-viewer/) and altitude above sea level from Google Earth (https://earth.google.com/web). Spatial visualization of the study area and sampling sites was performed using QGIS version 3.40.1-Bratislava. Administrative boundary data were obtained from the Natural Earth open-source database (https://www.naturalearthdata.com/). OpenStreetMap (OSM) data (https://planet.openstreetmap.org) rendered via the CARTO Voyager basemap (https://carto.com/basemaps/) were used as the background layer. OSM data are available under the Open Database License (ODbL), and the CARTO Voyager basemap is provided under the Creative Commons Attribution 3.0 license.

Statistical analyses were performed using chi-square tests to compare seroprevalence values among locations, following analytical practices used in comparable Toxoplasma sero-epidemiological research [30]. Associations between environmental variables and infection prevalence were explored using correlation-based approaches as described in earlier studies investigating climatic influences on *T. gondii* transmission in livestock populations [30].

This study relied on livestock sera collected as part of routine regional surveillance activities and not through a prospectively designed sampling scheme. Therefore, a priori sample size calculations based on expected seroprevalence, desired precision, power, and confidence level could not be performed. The number of animals sampled per locality reflected herd’s availability and accessibility of farms. As a result, the sample size across localities was uneven, and some locations contributed fewer than five animals.

To avoid unstable prevalence estimates, localities with fewer than five animals per species were excluded from locality-level prevalence calculations and from analyses that compared seroprevalence across geographic gradients. However, individual observations from all animals were retained in the logistic regression models, which evaluate seropositivity at the individual level rather than relying on aggregated prevalence values.

Pigs were sampled in only a single area, and no environmental variability could be assessed. For this reason, pigs were excluded from all regression analyses and environmental modeling, and only their crude seroprevalence is reported descriptively. The limited representation of pigs is acknowledged as a methodological limitation of the study.

Environmental effects on *T. gondii* seropositivity were evaluated using a two-stage analytical approach. First, exploratory analyses were conducted to assess the distributional properties, collinearity structure, and biological plausibility of all available environmental variables, including temperature, elevation, rainfall (mm), rainfall intensity (mm/day), and relative humidity. Because preliminary descriptive statistics and cross-tabulations showed that seroprevalence differed strongly between host species, all subsequent inferential analyses were performed separately for cattle and sheep.

Before model fitting, each continuous environmental variable was examined for normality, extreme values, and mutual correlations. Rainfall (mm), rainfall intensity (mm/day), and humidity (%) showed very limited variation across the sampled locations. These three variables were highly clustered around narrow ranges, reflecting the relatively homogeneous climatic conditions within sampled provinces during the study period. Visual inspection of scatterplots and coefficient-of-variation indices indicated that the effective dynamic range of these variables was too small to meaningfully capture biologically relevant gradients in oocyst survival or environmental contamination. In addition, rainfall and humidity were strongly intercorrelated with each other (r > 0.85), raising concerns about multicollinearity if included simultaneously in regression models.

Based on these diagnostics, rainfall and humidity were excluded from multivariable models, as their restricted variability and collinearity made them unsuitable for estimating species-specific effects. In contrast, temperature and elevation displayed substantially wider variation across sampling sites, showed no critical collinearity, and corresponded to meaningful ecological gradients in Armenia. Therefore, these two predictors were selected for formal modelling.

Elevation was rescaled as “Alt100” (elevation divided by 100 m) to improve interpretability of regression coefficients and numerical stability. Both temperature and Alt100 were included in their linear and quadratic forms (Temp, Temp^2^, Alt100, Alt100^2^) to allow detection of potential non-linear exposure patterns, such as mid-elevation minima or thermal thresholds affecting oocyst viability.

For each host species, *T. gondii* seropositivity (ELISA result: 0/1) was analyzed using logistic regression within a generalized linear model (GLM) framework. A null model containing only the intercept was compared with a full model including environmental predictors using a likelihood-ratio χ² test. Model performance and relative fit were evaluated using the Akaike Information Criterion (AIC) and several pseudo-R^2^ statistics (McFadden’s, Nagelkerke’s, and Tjur’s R^2^).

For each species, multivariable logistic regression models were fitted, and we report adjusted odds ratios (AORs) with 95% confidence intervals, obtained by exponentiating the regression coefficients (β) while adjusting for all other covariates in the model.

Because the ELISA outcome was binary, model-predicted probabilities were generated from the fitted GLMs to provide a continuous estimate of seropositivity across environmental gradients. These predicted probabilities were used for visualization, as they allow clearer representation of nonlinear or weak associations than raw binary values. Marginal predicted probabilities were computed across the observed range of each predictor and plotted using LOESS-smoothed curves.

All analyses were conducted in JASP (version 0.95.4.0), and model code and diagnostics are provided in the supplementary materials.

## Results

As a result of serological screening 312 out of 1,996 examined samples were positive for *T. gondii* antibodies including cattle (10.1% (112/1,112; 95% CI: 8.4–12.0)), sheep (22.5% (196/870; 95% CI: 19.8–25.5)), and pigs (28.6% (4/14; 95% CI: 8.4–58.1)).

Seroprevalence differed markedly between the two livestock species. After excluding pigs due to insufficient sample size, sheep showed a substantially higher seropositivity rate (22.5% (196/870; 95% CI: 19.8–25.5)) compared with cattle (10.1% (112/1,112; 95% CI: 8.4–12.0)). A Pearson’s chi-square test demonstrated a strong and statistically significant association between host species and *T. gondii* infection status (χ² = 58.31, df = 1, p < 0.001).

A total of 1,112 cattle and 870 sheep were included in the final dataset and analyzed separately because preliminary inspections revealed substantial differences in seroprevalence patterns between host species. The binary outcome (ELISA positive vs. negative) was modeled using logistic regression and generalized linear models with a logit link. Temperature and elevation were included as continuous predictors, with both linear and quadratic terms (Alt100 and Alt100²; Temp and Temp²) to capture possible non-linear environmental effects.

### Environmental predictors in cattle

For cattle, the generalized linear model that included all environmental predictors did not significantly improve model fit compared with the null model. The likelihood ratio test between the full model and the intercept-only model yielded a Δχ² value of 6.52 with 4 degrees of freedom (p = 0.164). Model fit indices further confirmed the limited explanatory power of the environmental variables: McFadden’s pseudo-R² was 0.009, Nagelkerke’s R² was 0.012, and Tjur’s R² was 0.006, all indicating that the included predictors explained only a very small fraction of the variability in *Toxoplasma gondii* seropositivity among cattle.

Regression coefficients likewise demonstrated no statistically significant associations between seropositivity and the environmental variables (Table 1).

**Table 1:** Multivariable logistic regression model of predictors of *T. gondii* seropositivity in cattle.

Predictor	Estimate (β)	SE	Adjusted Odds Ratio (AOR)	95% CI for AOR	p-value
Temperature	0.387	0.394	1.47	0.68–3.19	0.326
Alt100	0.168	0.286	1.18	0.68–2.07	0.557
Alt100^2^	−0.004	0.010	1.00	0.98–1.02	0.711
Temp^2^	−0.014	0.026	0.99	0.94–1.04	0.580

SE, standard error; AOR, adjusted odds ratio.

The insignificance of both linear and quadratic terms suggests that, within the sampled cattle populations, neither temperature nor elevation exerted measurable influence on the likelihood of *T. gondii* exposure.

Estimated marginal means also reflected the weak and uncertain relationship between altitude and infection probability. Predicted seropositivity ranged from approximately 0.05 at Alt100 = 10.6 (roughly 1,060 m) to 0.18 at Alt100 = 18.6 (1,860 m), but confidence intervals were broad, indicating high uncertainty around these estimates (Table 2).

**Table 2:** Estimated marginal means (cattle).

Altitude (Alt100)	Predicted probability	SE	95% CI
10.6	0.053	0.058	0.006–0.349
14.61	0.098	0.009	0.082–0.118
18.63	0.176	0.167	0.022–0.671

SE, standard error.

**Fig 2:**
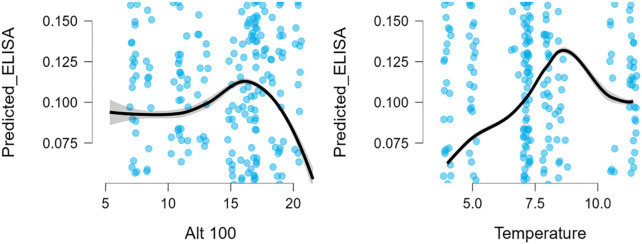
Cattle: LOESS-smoothed predicted probability curves for Temperature and Altitude.

LOESS-smoothed prediction plots produced only shallow, inconsistent curves across temperature and altitude ranges, with no clear ecological trends (Fig 2).

Taken together, these findings indicate that cattle seropositivity in this dataset is largely unaffected by the environmental factors considered.

### Environmental predictors in sheep

In contrast to the cattle results, the logistic regression model for sheep showed a strong and statistically robust association between seropositivity and environmental predictors. The full model significantly improved upon the null model, with Δχ² = 85.98 (df = 4, p < 0.001). Model performance indices were substantially higher than in cattle, with McFadden’s R² = 0.093, Nagelkerke’s R² = 0.143, and Tjur’s R² = 0.101, indicating that the environmental variables explained a meaningful portion of the variation in *T. gondii* exposure in sheep.

Among the individual predictors, elevation emerged as the dominant driver of infection probability. The linear effect of altitude (Alt100) was significantly negative, indicating that seropositivity decreased with increasing elevation. At the same time, the quadratic term for altitude was significantly positive, suggesting a non-linear pattern in which the probability of seropositivity declines steadily across low to mid-elevations but then rises slightly at high elevations. This combination of effects produces a U-shaped environmental response, with the lowest predicted seroprevalence occurring at mid-altitudes. Temperature did not show a significant effect in sheep, neither in its linear form nor as a quadratic term, suggesting that temperature gradients across the sampling range did not play a major role in shaping *T. gondii* exposure among sheep (Table 3).

**Table 3:** Multivariable logistic regression model of predictors of *Toxoplasma gondii* seropositivity in sheep.

Predictor	Estimate (β)	SE	Adjusted Odds Ratio (AOR)	95% CI for AOR	p-value
Temperature	−0.040	0.214	0.96	0.63–1.46	0.853
Alt100	−0.498	0.148	0.61	0.45–0.81	<0.001
Alt100^2^	0.021	0.005	1.02	1.01–1.03	<0.001
Temp^2^	0.014	0.010	1.01	0.99–1.04	0.161

SE, standard error; AOR, adjusted odds ratio.

Estimated marginal means further illustrate the strength of the altitude effect. Predicted seropositivity was highest at lower altitudes, reaching approximately 0.70 at Alt100 ≈ 9.9 (roughly 990 m). Seropositivity then declined steeply toward mid-elevation levels, with a predicted probability of 0.20 near Alt100 ≈ 14.3 (1,430 m). At higher elevations (Alt100 ≈ 18.8; 1,880 m), the predicted probability dropped further to around 0.03, though confidence intervals widened slightly due to smaller sample densities at the highest altitudes (Table 4).

**Table 4:** Estimated marginal means (sheep).

Altitude (Alt100)	Predicted probability	SE	95% CI
9.89	0.697	0.137	0.391–0.892
14.35	0.200	0.015	0.173–0.231
18.81	0.027	0.018	0.007–0.094

SE, Standard error.

These results indicate a strong and non-linear dependence of *T. gondii* exposure on elevation in sheep, which contrasts sharply with the absence of identifiable environmental effects in cattle.

**Fig 3:**
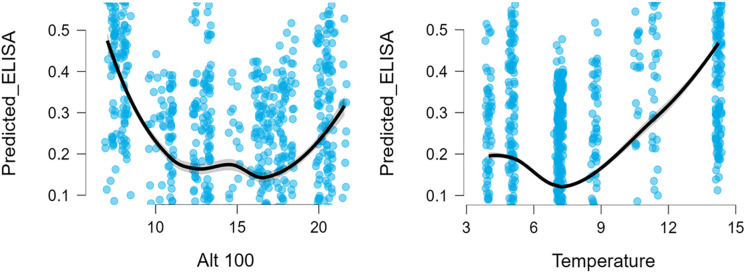
Sheep: Strong non-linear effects of altitude on predicted probability. A minimum at mid-altitudes and increases toward both low and high ends.

LOESS curves for sheep clearly visualized the non-linear altitude response: a pronounced decline in seropositivity across the mid-altitude range and a modest upturn at the uppermost elevations (Fig 3).

## Discussion

In the present study, the overall seroprevalence of *T. gondii* in livestock from northern Armenia was 15.6% (308/1,982; 95% CI: 14.0–17.2), consistent with levels reported from several regions [31–33]. Although seroprevalence was higher in sheep than in cattle, the magnitude of this difference was modest and cannot be interpreted as a temporal increase, especially as the studies conducted 35 years ago differed in sampling design, diagnostic methods, and environmental conditions. Therefore, no claims about trends over time are made.

The importance of public health of our findings lies primarily in the potential exposure risk through consumption of undercooked meat. In many countries, the consumption of insufficiently cooked meat from small ruminants is considered an important source of *T. gondii* infection for humans [7,34]. Our data suggest that meat from sheep – particularly in areas where seroprevalence is higher—may contribute to the risk of foodborne transmission if consumed undercooked.

Like findings reported in Pakistan, we observed that environmental parameters influenced spatial variation in *T. gondii* exposure, supporting the role of climatic conditions in shaping transmission risk in grazing animals [30].

### Sheep

Sheep and goats are considered as a major risk factor for human infection in many countries. The overall worldwide seroprevalence of *T. gondii* infection was 33.86% in sheep, with significant variation across geographical locations [12]. Among sheep from various European countries, the seroprevalence of *T. gondii* varies: 17.2% in Latvia [35], 53.5% in Romania [36], 74% in Great Britain [37], 46.5% in Spain [38], 87.4% in Belgium [39].

In Southern Iran, regions with high humidity reported a 10.71% prevalence of toxoplasmosis in sheep, as detected by molecular methods. In contrast, no positive cases were found in areas characterized by higher temperatures and lower humidity [40]. In neighboring Turkey, the lowest seropositivity rate in previous studies from 1976 to 2014 was found in Konya province with 13%, and the highest in Afyon province with 99.19% [41,42]. As opposed to our results, in a recent study (2015), the lowest *T. gondii* seroprevalence was found in Erzurum (4.58%), which is the highest province of Turkey (1860 m above sea level). The lower seroprevalence of *T. gondii* in Erzurum compared to other regions of Turkey is probably due to its climate and geographical conditions (short and arid summers and long, snowy, cold winters) [43].

In Nepal, the overall seroprevalence of *T. gondii* in sheep is 36.17%. The highest seroprevalence in sheep was detected at a low altitude with a hot and humid climate (Pokhara 32.94% and Chitwan 57.65%), and the lowest seroprevalence was found at high altitude with the arid environment (Jumla) [44]. Similar findings were found in Mexico, where at low altitude and semi-intensive sheep farming was detected the highest prevalence of *T. gondii* [45].

In sheep, *T. gondii* seropositivity showed a pronounced and statistically robust association with altitude. Both the linear altitude predictor (Alt100) and its quadratic term were significant, indicating a non-linear elevation effect. Seropositivity was highest at mid-range elevations (~1,000–1,500 m) and declined at higher altitudes. This pattern likely reflects the combined influence of land use, cat density, and oocyst survival, rather than altitude itself acting as a biological driver. Interestingly, in contrast to our findings, in Oaxaca State, Mexico, sheep raised in temperate climates at elevations of 1,560–1,600 m indicate a significantly higher seroprevalence of *T. gondii* infection (29.8%) compared to those raised in semiarid and warm-humid climates at elevations of 1,020–1,080 m (7.1%) [46].

At mid-altitudes in northern Armenia, sheep grazing typically occurs near densely inhabited villages, where domestic and semi-feral cats are common. These grazing systems create persistent environmental contamination through oocyst shedding [47,48].

In contrast, pastures at high altitude (>1,900 m) are cooler, less humid, and more sparsely populated by both livestock and cats, reducing opportunities for exposure.

Temperature and humidity dependent oocyst survival provides a mechanistic explanation. *T. gondii* oocysts remain viable longest in moist, moderate-temperature soils (optimal 10–25°C), while extreme cold or dryness reduces persistence [49–52].

These conditions match mid-altitude Armenian grazing zones but not higher alpine pastures. Thus, the observed pattern likely reflects environment-mediated oocyst persistence and grazing ecology, rather than altitudinal physiology. Sheep are free-grazing animals, often feeding close to the ground and drinking from open water sources [53]. Our results align with this ecological expectation. sheep consistently showed higher seropositivity than cattle in shared locations of northern Armenia. The stronger altitude signal in sheep compared with cattle may therefore reflect grazing practices, rather than inherent host susceptibility.

The quadratic relationship suggests that infection likelihood increases up to a certain elevation, then decreases. This pattern is demonstrated because of urban zone densities and accordingly cat density decreases at high elevations [54,55], reducing environmental contamination, oocyst persistence declines at cold, dry highlands [49,52], sheep grazing is more seasonal at high altitudes in Armenia, reducing exposure duration.

These ecological processes explain the non-linear altitude pattern found in our study.

The elevation-dependent risk in sheep indicates that exposure is shaped by local grazing management and proximity to human settlements, rather than broad climatic variables. This highlights the need for micro-ecological sampling (soil and water testing), cat population monitoring along elevation gradients, targeted educational interventions for shepherds at high-risk mid-altitude farms.

Studies revealed a relationship between *T. gondii* prevalence and temperature and rain in France. Oocyst survival increases in moist conditions during longer periods of hot weather, and as a result, the risk of acquiring an infection is enhanced [56,57].

In our study, in contrast to altitude, temperature was not significantly associated with seropositivity in the sheep-specific regression. This does not contradict existing knowledge. Across many temperate regions, climatic effects on *T. gondii* transmission may become detectable only in long-term, fine-scale datasets, such as monthly environmental records linked to soil moisture and local habitat conditions. In our study, the environmental dataset consisted of point-based, satellite-derived annual averages, which are relatively coarse and may not capture micro-habitat differences that meaningfully influence oocyst survival. In addition, previous studies have shown that seasonal precipitation can shape the environmental transport of oocysts by influencing runoff and river flow, thereby contributing to water-borne toxoplasmosis and coastal contamination with this protozoan parasite [58–60].

### Pig

A recent risk assessment report ranked the combination of *Toxoplasma* and pork as the second highest out of ten pathogen-food pairings in terms of risk [61]. The global pooled seroprevalence of *T. gondii* in pigs was estimated at 19%, with Europe having the lowest rate (13%) and Africa and North America both showing the highest rates (25%).

Seropositive pigs were identified in the study. However, because pig samples were available from only one locality (S1 Table), the observed seroprevalence of 28.6% (4/14; 95% CI: 8.4–58.1) cannot be considered representative of the examined region. Therefore, these data should be interpreted cautiously, and no broader conclusions regarding environmental patterns or public health risk can be drawn from this species in the present study.

### Cattle

Bovines are a major source of meat for humans worldwide. However, the clinical symptoms of toxoplasmosis in bovines are generally milder or unknown compared to those in sheep, goats, and pigs [31]. The global pooled seroprevalence of *T. gondii* among bovines is 17.91%, of which the seroprevalence among cattle is 16.94% [31].

In our region, the seroprevalence of toxoplasmosis in cattle in Iran for 30 years was 18.1% (9.5%−28.2%) [62]. In Turkey, the seroprevalence of *T. gondii* among cattle ranges from 2.6 to 66.03% [63].

In cattle, logistic regression did not identify significant associations between seropositivity and any of the environmental variables examined. This is consistent with the biological characteristics of cattle and their exposure pathways, as cattle are widely recognized as poor intermediate hosts for *T. gondii* [64].

The lower seropositivity in cattle compared with sheep may also be related to differences in grazing behaviour, since cattle typically feed on the upper parts of grass and browse higher vegetation, whereas sheep graze closer to the ground and are therefore more likely to ingest sporulated oocysts from contaminated soil [1].

The absence of statistically significant predictors in our cattle model, therefore, likely reflects low exposure frequency, reduced biological susceptibility, and distinct foraging behavior, rather than an analytical shortcoming. This interpretation is further supported by the consistently low seroprevalence observed across cattle populations in the study region [65].

Although cattle meat is generally considered less important for transmission to humans compared with small ruminant or pig meat, the detection of seropositive animals indicates that exposure does occur [66–68]. However, given the low infection levels, cattle are unlikely to be a major reservoir for *T. gondii* transmission in northern Armenia.

### Limitations

This study has several limitations that should be considered when interpreting the findings:

- Demographic information on individual animals (age, sex, reproductive status, breed, and husbandry details) was not available, preventing assessment of well-established risk factors for *T. gondii* exposure and limiting the ability to adjust for important confounders.
- Although sampling was conducted across 73 localities, some of these included very small numbers of sampled animals per species.
- Pigs were sampled in only a single area, preventing meaningful epidemiological comparisons and limiting conclusions for this species.
- While environmental variables were analyzed at broad spatial and temporal scales, fine-scale farm-level practices, such as cat density, feed storage, biosecurity measures, were not available but likely contribute substantially to species-specific exposure differences.
- The study relied exclusively on serology, which detects past exposure but cannot distinguish recent from chronic infections or confirm the presence of viable tissue cysts in meat.

So future studies incorporating molecular diagnostics and more detailed metadata would allow a more comprehensive assessment of infection dynamics and associated risk factors.

## Conclusion

This study provides updated evidence on *Toxoplasma gondii* seroprevalence in northern Armenian livestock, showing consistently higher infection levels in sheep compared with cattle. Environmental modelling revealed that altitude was the only significant predictor of seropositivity, and only in sheep, suggesting species-specific exposure pathways. While pigs were sampled in only one locality and were therefore excluded from statistical comparisons, their seropositivity indicates that swine may also contribute to human exposure where consumed undercooked.

These findings underscore the need for expanded surveillance, including larger geographic coverage and complementary molecular approaches capable of detecting active infection and confirming the presence of viable tissue cysts. Improved understanding of how environmental and management factors differentially influence infection risk in livestock will support the development of more effective food-safety and toxoplasmosis control strategies.

## Supporting information

S1 Table The number of sampled animals per locality, ELISA results, and means of environmental variables. (CSV)
